# ALDOC promotes neuroblastoma progression and modulates sensitivity to chemotherapy drugs by enhancing aerobic glycolysis

**DOI:** 10.3389/fimmu.2025.1573815

**Published:** 2025-04-17

**Authors:** Yunpeng Chen, Haixia Zhu, Yishu Luo, Tianyue Xie, Youyang Hu, Zhiwei Yan, Weichao Ji, YaXuan Wang, Qiyou Yin, Hua Xian

**Affiliations:** ^1^ School of Medicine, Nantong University, Nantong, China; ^2^ Cancer Research Center Nantong, Nantong Tumor Hospital, Nantong, Jiangsu, China; ^3^ Department of Endocrinology, Affiliated Hospital of Nantong University, Nantong, China; ^4^ Department of Cardiothoracic Surgery, Affiliated Hospital of Nantong University, Nantong, Jiangsu, China; ^5^ Department of Urology, Nantong Tumor Hospital, Nantong, China; ^6^ Department of Paediatric Surgery, Affiliated Hospital of Nantong University, Nantong, Jiangsu, China

**Keywords:** aerobic glycolysis, neuroblastoma, ALDOC, MYCN, drug sensitivity

## Abstract

**Introduction:**

Neuroblastoma (NB), a malignant extracranial solid tumor originating from the sympathetic nervous system, exhibits poor prognosis in high-risk cases, with a 5-year overall survival rate below 50%. Glycolysis has been implicated in NB pathogenesis, and targeting glycolysis-related pathways shows therapeutic potential. This study investigates the role of the glycolysis-associated gene ALDOC in NB pathogenesis and its impact on chemotherapy sensitivity.

**Methods:**

Transcriptomic data from NB patients were analyzed to identify ALDOC as an independent risk factor for high-risk NB. Protein expression levels of ALDOC were assessed in NB cells versus normal cells using immunoblotting. Functional experiments, including proliferation and migration assays, were conducted in ALDOC-interfered NB cell lines. Glycolytic activity was evaluated by measuring glucose uptake, lactate production, and ATP generation. Additionally, the sensitivity of ALDOC-downregulated NB cells to cisplatin and cyclophosphamide was tested to explore its role in chemotherapy response.

**Results:**

ALDOC was identified as a high-risk prognostic marker in NB, with elevated protein expression in NB cells compared to normal controls. Silencing ALDOC significantly inhibited NB cell proliferation and migration. Glycolytic activity was markedly reduced in ALDOC-downregulated cells, evidenced by decreased glucose uptake, lactate production, and ATP levels. Furthermore, ALDOC suppression enhanced NB cell sensitivity to cisplatin and cyclophosphamide, suggesting a glycolysis-dependent mechanism underlying chemotherapy resistance.

**Discussion:**

Our findings highlight ALDOC as a critical driver of NB progression through glycolysis acceleration, with implications for therapeutic targeting. The observed increase in chemotherapy sensitivity upon ALDOC inhibition underscores its potential as a biomarker for treatment optimization. However, the complexity of glycolysis regulation, involving multiple genes and pathways, necessitates further mechanistic studies to clarify ALDOC’s specific role. Despite this limitation, our work emphasizes the importance of aerobic glycolysis in NB pathogenesis and provides a foundation for developing novel therapeutic strategies targeting ALDOC or associated pathways. Future research should explore interactions between ALDOC and other glycolytic regulators to refine combinatorial treatment approaches.

## 1 Introduction

Neuroblastoma (NB) is the most common malignant extracranial solid tumor in infants and young children (1, 2), and typically originates from the adrenal glands (3, 4). NB often presents within 1-2 years after birth, with a median age at diagnosis of 17 months (2, 5). Due to its insidious onset, rapid progression, and early tendency for distant metastasis, the majority of patients are diagnosed at a high-risk stage. Notably, researchers have reported a significant disparity between the incidence and mortality rates of NB (6). Although NB accounts for 6%-10% of all childhood malignancies, its mortality rate can reach 15% (1, 7). Benefiting from multicenter clinical trials conducted internationally and the implementation of multidisciplinary, multimodal therapies, the 5-year survival rate for NB patients has reached approximately 75%. However, the surprising aspect of this improvement lies in the substantial increase in cure rates for low- and intermediate-risk groups, with survival rates approaching 100%. However, for high-risk NB patients, the 5-year survival rate has risen to only approximately 50% (4, 8–12). Therefore, the identification of biomarkers for high-risk NB and genes associated with drug resistance in NB patients is highly important for the development of new treatment strategies.

Tumor cells tend to rely on glycolysis to acquire energy. Studies have shown that during the malignant proliferation of tumor cells, they exhibit a sustained high demand for energy. However, due to their lack of metabolic flexibility, these enzymes switch from oxidative phosphorylation (OXPHOS) to glycolysis to obtain energy (13). This metabolic reprogramming is not restricted by oxygen levels; even in the presence of abundant oxygen, tumor cells primarily utilize glycolysis to generate energy. Moreover, this pathway also provides various biomolecules necessary for tumor cell growth and synthesis. This phenomenon, first proposed by Otto Warburg, is hence referred to as the “Warburg effect” or aerobic glycolysis (14–16). Recent research has focused on investigating individual genes. In contrast, we employed a bioinformatics approach to analyze transcriptomic big data to screen for prognostic genes. Compared with direct studies of single genes, our method is more comprehensive and universal. Using such approaches, researchers have detected the upregulated expression of various key glycolytic enzymes, such as hexokinase 2 (HK2), phosphoglycerate kinase 1 (PGK1), aldolase, and lactate dehydrogenase A (LDHA), in multiple tumor cell types (17–19).

Relevant studies have confirmed that the level of glycolysis affects the sensitivity of tumor cells to drugs (20). Research on drug-resistant tumor types has revealed increased levels of aerobic glycolysis in resistant strains (17). These findings support the close association between glycolysis and chemotherapy resistance in tumor cells. Since chemotherapy drugs often kill tumor cells by inducing oxidative stress, the glycolysis of tumor cells can generate sufficient NADPH to maintain glutathione (GSH) levels and alleviate tumor cell sensitivity to chemotherapy drugs. GSH is an intracellular antioxidant that protects cells from oxidative damage by clearing oxidative stressors, thus enabling tumor cells to evade the effects of chemotherapy drugs and leading to chemotherapy resistance (21, 22). Additionally, the emergence of drug resistance, especially multidrug resistance (MDR), is one of the important reasons for poor treatment outcomes or recurrence in patients with various advanced malignant tumors (23, 24). Considering that NB relies on aerobic glycolysis as a means of obtaining energy, targeting this metabolic pathway may represent a potentially advantageous therapeutic strategy for NB treatment.

Emerging evidence from multiple studies has established a positive correlation between aldolase C (ALDOC) expression and cancer progression across various malignancies. ALDOC demonstrates significant overexpression in gastric cancer (GC) (25) and colorectal cancer (CRC) (26), where it directly promotes tumor proliferation, invasion, and metastasis through glycolytic reprogramming – a hallmark of the Warburg effect. Beyond metabolic regulation, ALDOC orchestrates tumor microenvironment remodeling by modulating immune cell infiltration (particularly macrophage differentiation) and upregulating immunosuppressive molecules. Mechanistically, ALDOC confers chemoresistance through dual mechanisms: metabolic adaptation (enhanced lactate production) and cross-talk with critical signaling pathways (HIF-1α and Akt) (27). Clinical correlations reveal that GC patients with ALDOC overexpression exhibit poor response to conventional chemotherapy and significantly reduced survival rates.

While these findings establish ALDOC’s multifaceted role in GC and CRC pathogenesis, its functional significance in NB remains unexplored. This study aims to investigate the role of aerobic glycolysis in the malignant progression of NB and elucidate its associated molecular mechanisms. Our preliminary bioinformatics analysis has identified a significant correlation between ALDOC expression levels and prognosis in high-risk NB patients. As a crucial regulatory protein in intracellular glycolysis, the specific functions and underlying molecular mechanisms of ALDOC in NB remain largely unexplored. This research seeks to systematically examine the association between ALDOC-mediated aerobic glycolysis and NB pathogenesis, while further evaluating its potential impact on the therapeutic efficacy of conventional chemotherapeutic agents (cisplatin and cyclophosphamide) in NB treatment. The findings are expected to provide novel therapeutic targets and strategic insights for NB management in the era of precision medicine.

## 2 Materials and methods

### 2.1 Cell lines and culture

The cell lines used for this study included five neuroblastoma cell lines. The IMR-32 and SH-SY5Y cell lines were purchased from Qingqi Biotechnology Development Co., Ltd. (Shanghai, China), and the SK-N-SH, SK-N-AS, and SK-N-BE(2) cell lines were obtained from Jiangsu Ads Biomedical Technology Co., Ltd. (Jiangsu, China), and all the cell lines were authenticated by a short tandem repeat (STR) analysis. Control cell (293T cell) were obtained from the Central Laboratory of Nantong Tumor Hospital. All the cell lines were cultured in Dulbecco’s modified Eagle medium (DMEM) supplemented with 10% fetal bovine serum and 1% penicillin-streptomycin (Gibco), and maintained at 37°C in a humidified atmosphere with 5% CO_2_. Note: The certificates of authentication for various neuroblastoma cell lines can be found in **Supplementary Material**.

### 2.2 Lentivirus sequence information

We utilized lentivirus to construct cell vectors for the knockdown and overexpression of the ALDOC gene. The knockdown lentiviral vector is named GV493, and its component sequence is: hU6-MCS-CBh-gcGFP-IRES-puromycin. The insert sequence of negative control virus is: TTCTCCGAACGTGTCACGT. The specific sequences for the three shRNA vectors are as follows: (115917-1: GCAGCACAGTCACTCTACATT; 115918-1: CTATTGTGGAACCTGAAATAT; 115919-1: CGACCTCAAACGTTGTCAGTA). For the overexpression group, the lentiviral vector is named GV492, and its component sequence is: Ubi-MCS-3FLAG-CBh-gcGFP-IRES-puromycin. The cloning sites of negative control virus is: BamHI/AgeI. The specific sequences for the overexpression vector are as follows:(85945-2-p1: AGGTCGACTCTAGAGGATCCCGCCACCATGCCTCACTCGTAC; 85945-2-p2: TCCTTGTAGTCCATACCGTAGGCATGGTTGGCAATGTAGAG).

### 2.2 Downloading sequencing data for neuroblastoma

The mRNA sequencing data for neuroblastoma samples were obtained from the GEO database (GSE49710) and used as the training set. The clinical information related to GSE49710 was sourced from GSE62564 (which provides supplementary explanations for GSE49710). The data for the validation set were downloaded and organized from the Target database. Detailed information is summarized in **Table 1**: Summary of the clinical information of the two neuroblastoma datasets. Transcriptome and proteome sequencing of 15 neuroblastoma clinical samples in this study was conducted by Spectra Biotech.

**Table 1:** Clinical characteristics of the two cohorts (GSE49710 and Target).

Characteristics	Training cohort	Validation cohort	P value
Data sources	GSE49710	Target	
n	498	153	
Age, median (IQR)	444.5 (165.25, 1008.5)	1064 (659, 1661)	< 0.001
Gender, n (%)			0.794
Male	287 (44.1%)	90 (13.8%)	
Female	211 (32.4%)	63 (9.7%)	
INSS Stage, n (%)			< 0.001
Stage 4	183 (28.1%)	125 (19.2%)	
Stage 2	78 (12%)	0 (0%)	
Stage 4s	53 (8.1%)	21 (3.2%)	
Stage 3	63 (9.7%)	6 (0.9%)	
Stage 1	121 (18.6%)	0 (0%)	
Stage 2b	0 (0%)	1 (0.2%)	
MYCN Statue, n (%)			0.654
Not Amplified	401 (61.6%)	119 (18.3%)	
Amplified	92 (14.1%)	33 (5.1%)	
Unknown	5 (0.8%)	1 (0.2%)	
Statue, n (%)			< 0.001
Dead	105 (16.1%)	81 (12.4%)	
Alive	393 (60.4%)	72 (11.1%)	
Overall Survival Time in Days, median (IQR)	2006.5 (1101, 3191.5)	1636 (768, 3264)	0.102

### 2.3 Western blot analysis

Cell lysis buffer was used to extract total cellular proteins, and the protein concentration in the cell lysates was measured using a BCA protein assay kit. Equal amounts of protein were subsequently subjected to Western blot analysis. The membranes were probed with antibodies against ALDOC and MYCN, followed by an incubation with the corresponding secondary antibodies. The chemiluminescent signal was detected using a highly sensitive ECL Western Blotting Substrate (Tanon, China) and visualized using a protein imaging system (Bio-Rad, USA).

### 2.4 CCK8 assay

The cells from each treatment group were seeded in a 96-well plate (Corning, USA) at a density of 3×10^3^ cells per well. After an incubation for 12 h, 24 h, 36 h, or 48 h, 100 μl of preprepared CCK8 assay reagent (Beyotime, China) was added to each well. The plate was then incubated at 37°C for 2 h in a cell culture incubator. The absorbance at 450 nm was measured using a microplate reader (Thermo Fisher, USA).

### 2.5 Colony formation assay

Cells transfected with an ALDOC shRNA lentiviral vector or an ALDOC empty vector were seeded into 6-well plates (200 cells/well). The cells were subsequently incubated in a cell culture incubator for 2 weeks to allow colony formation. After 2 weeks, the cells in the 6-well plates were fixed and stained with 0.1% crystal violet. Excess dye was removed by washing with water, and the number of cell colonies on each plate was counted.

### 2.6 Transwell assay

The cells from each ALDOC treatment group were digested with trypsin, centrifuged, and resuspended in serum-free culture medium to a density of 5×10^5^ cells/ml. Subsequently, 200 μl of the cell suspension was seeded into wells containing 500 μl of complete culture medium and placed in an incubator for cultivation. After 24 h, the cells were fixed with 4% paraformaldehyde, stained with 0.1% crystal violet, rinsed with water to remove excess dye, gently wiped with a cotton swab to clean the inner surface of the wells, air-dried, photographed under a microscope, and counted using software.

### 2.7 Scratch assay

After stable transfection with lentiviruses, cells from the negative control group and the knockdown/overexpression group were digested with trypsin, centrifuged at 1200 rpm for 6 min, and resuspended in culture medium. Subsequently, 5×10^5^ cells were seeded uniformly in each well of a 6-well plate, with 3 replicate wells per group, for a total of 4 6-well plates. The plates were then placed in a cell culture incubator, with regular monitoring of cell growth. When the cell density was greater than 90%, a line was drawn vertically across each well using a pipette tip, and photographs were captured at 0 h, 24 h, and 48 h. Finally, data were analyzed using ImageJ software.

### 2.8 Lactic acid assay

The cells from each treatment group were seeded into culture dishes at a density of 3×10^5^ cells/well and then incubated in a cell culture incubator at 37°C with 5% CO_2_ for 24 h. After 24 h, 1 ml of medium from each treatment group was aspirated as the sample for subsequent experiments. The working solution and color reagent were prepared according to the instructions provided with each reagent. The samples were subsequently added to EP tubes followed by the addition of the enzyme working solution and color reagent. The mixture was incubated at 37°C for 10 min, and after the stop solution was added, the mixture was transferred to a 96-well plate and the absorbance at 530 nm was measured.

### 2.9 Glucose consumption assay

The cells from each treatment group were seeded into 6-well plates at a density of 2×10^5^ cells per well and incubated in a 37°C incubator for 24 h. After the incubation, the culture medium from each group of cells was collected to measure the glucose content. Standard samples and test samples were then transferred to PCR tubes, followed by the addition of glucose assay reagent (Beyotime, China). After thorough mixing, the samples were centrifuged to settle them at the bottom of the tube. The tubes were then heated at 95°C in the dark for 8 min and subsequently cooled to 4°C. After cooling, the tubes were centrifuged again to settle the sample at the bottom of the tube, and then, the sample was transferred to a 96-well plate to measure the absorbance at 630 nm.

### 2.10 ATP assay

The cells in each treatment group were seeded into culture dishes at a density of 3×10^5^ cells/well and then incubated in a cell culture incubator for 24 h. After 24 h, the cells were lysed and homogenized, and the protein concentration was measured. The assay reagents were prepared according to the instructions provided with the reagents, and each reagent was sequentially added as required for the reaction. Following the completion of the reaction, the reaction mixture was transferred to a 96-well plate, and the absorbance at 636 nm was measured. Statistical calculations were performed accordingly.

### 2.12 Drug sensitivity assay

Cells from different treatment groups, including the ALDOC-Control group, sh-ALDOC group, and OE-ALDOC group, which exhibited good adherence (over 90% confluency), were uniformly seeded into a 96-well plate at a density of 5000 cells/well. Seven concentration gradients were prepared, with each gradient comprising five replicate wells. The seeded plates were then incubated in a cell culture incubator for 24 h. After 24 h, the drugs were diluted in complete medium to predetermined concentrations and added to the corresponding wells of the 96-well plate. The plate was then incubated for an additional 24 h. After the incubation, a CCK8 assay was performed, and the absorbance of each well was measured. The cell viability at each concentration was subsequently calculated.

### 2.13 Statistical analysis

**Figure 1:**
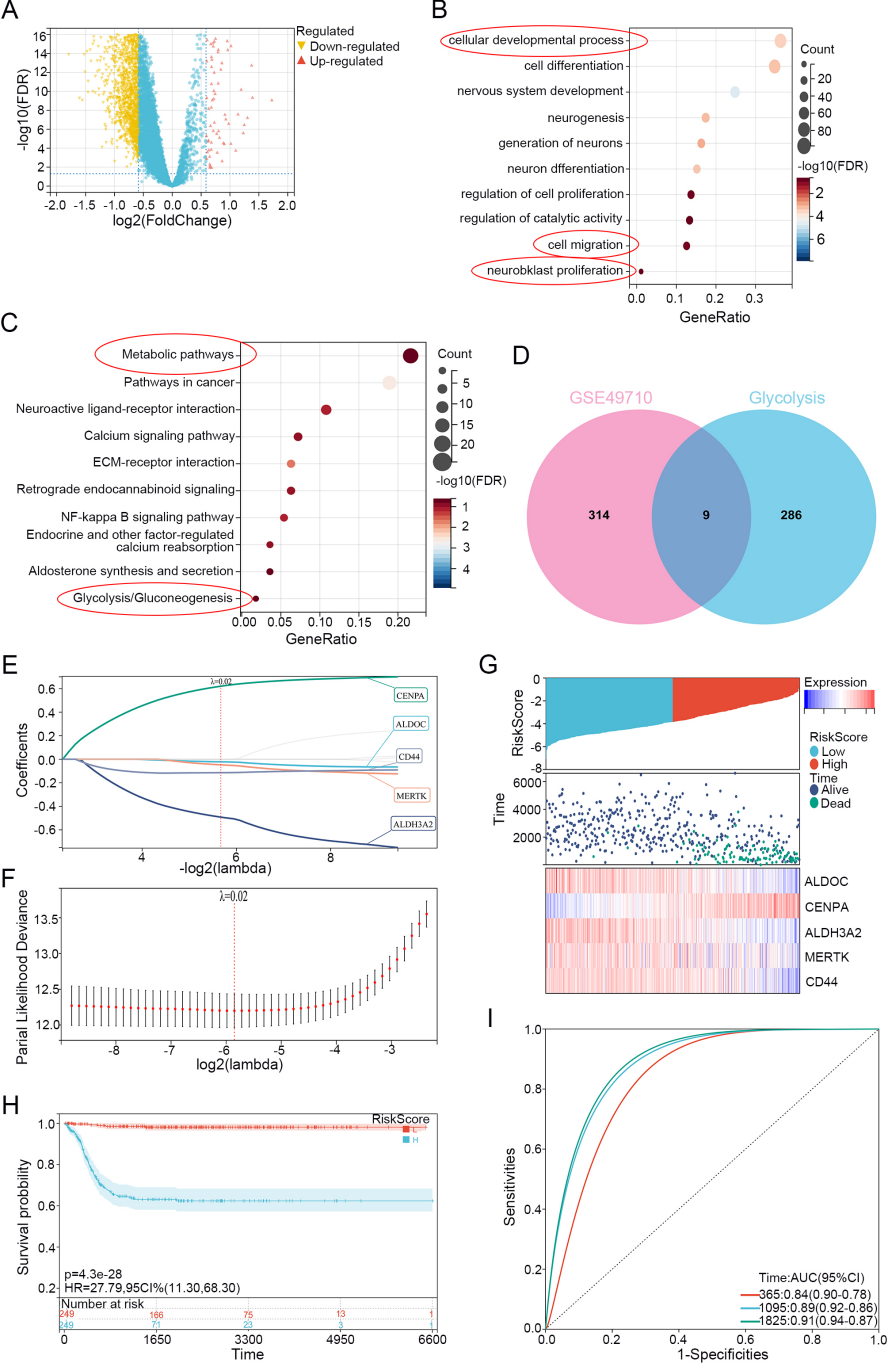
Neuroblastoma Glycolysis-Related Gene selection and Lasso model establishment. **(A)** Volcano plot of differential analysis using limma on dataset GSE49710 (Stage 4 vs. non-stage 4), with upregulated genes highlighted in red and downregulated genes highlighted in yellow. **(B)** GO enrichment analysis of differentially expressed genes. **(C)** KEGG enrichment analysis of differentially expressed genes. **(D)** Venn diagram showing the overlap between differentially expressed genes and genes related to glycolysis. **(E)** LASSO regression of differentially expressed genes related to glycolysis. **(F)** λ values in the established LASSO regression model. **(G)** Risk distribution, survival status, and heatmap of gene expression in the training cohort. **(H)** Overall survival curve based on the LASSO regression model's KM analysis. **(I)** ROC curves for 1, 3, and 5 years based on the LASSO regression model.

**Figure 2:**
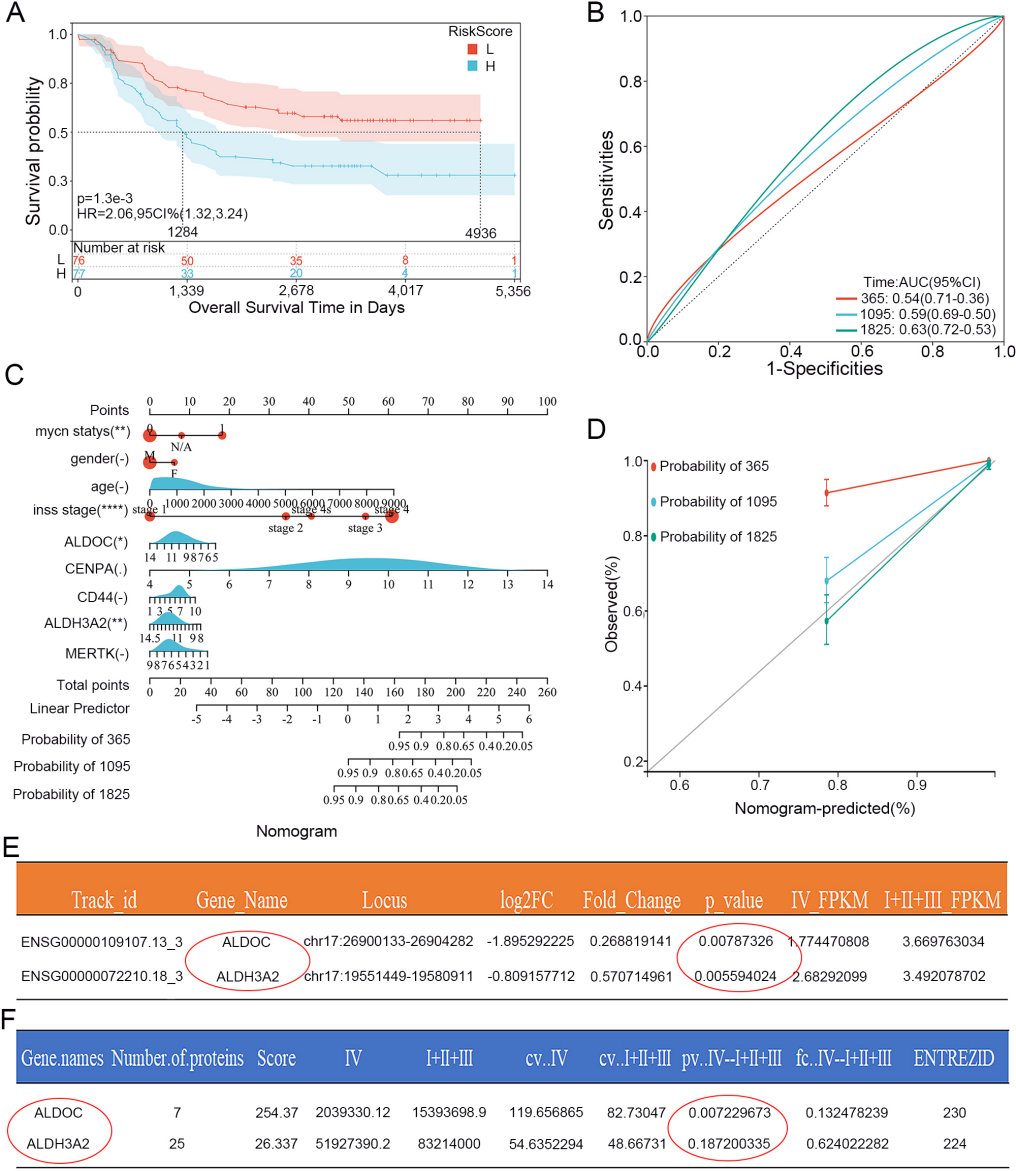
Validation of the Lasso prognostic model in the training set and determination of the independent risk factor ALDOC. **(A)** Kaplan-Meier curves of genes in the validation set from the Lasso prognostic model. **(B)** ROC curves for 1, 3, and 5 years of genes in the validation set from the Lasso prognostic model. **(C)** Nomogram plot based on genes and clinical characteristics from the Lasso regression model and the training set. **(D)** Calibration curves for 1, 3, and 5 years based on the Nomogram plot. **(E)** Statistical analysis of ALDOC and ALDH3A2 mRNA expression in 15 clinical samples sequencing results (Stage 4 vs. non-Stage 4). **(F)** Statistical analysis of ALDOC and ALDH3A2 protein expression in 15 clinical samples sequencing results (Stage 4 vs. non-Stage 4).

**Figure 3:**
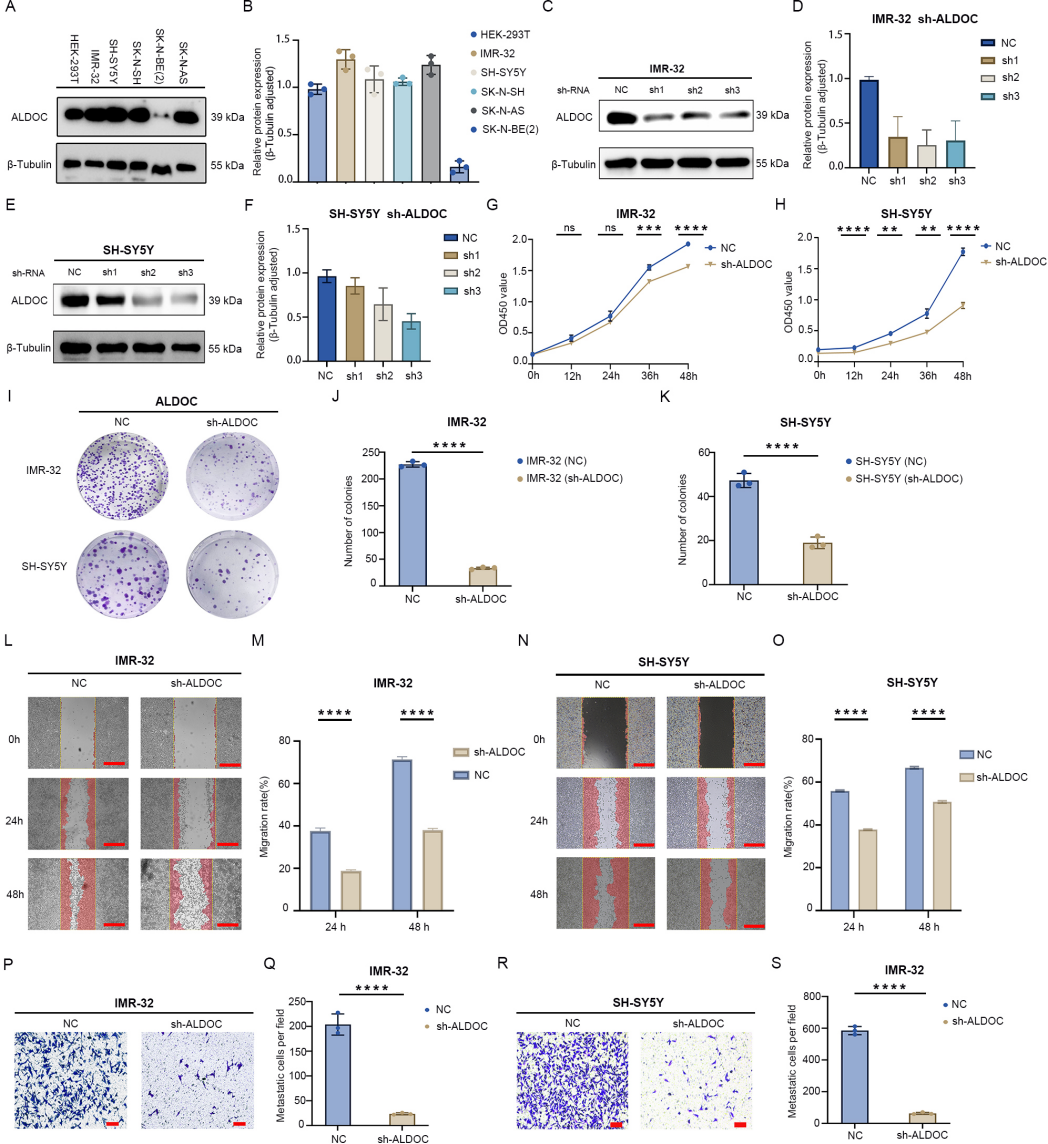
Downregulation of ALDOC expression inhibits neuroblastoma cell proliferation and migration. **(A)** Expression of ALDOC in control cells and neuroblastoma cell lines. **(B)** Statistical graph of protein expression levels. **(C)** Protein validation of ALDOC knockdown in IMR-32 cell line. **(D)** Statistical analysis of protein validation results of ALDOC knockdown in IMR-32 cell line. **(E)** Protein validation of ALDOC knockdown in SH-SY5Y cell line. **(F)** Statistical analysis of protein validation results of ALDOC knockdown in SH-SY5Y cell line. **(G, H)** CCK-8 proliferation assay results of ALDOC knockdown in IMR-32 cell line/SH-SY5Y cell line compared to control group. **(I)** Cell cloning assay results of ALDOC knockdown groups compared to control group. **(J, K)** Statistical analysis of cell clone colonies in IMR-32 cells/SH-SY5Y cells with ALDOC knockdown group compared to control group. **(L)** Scratch assay results of ALDOC knockdown compared to control group in IMR-32 cell lines. **(M)** Statistical analysis of cell migration rate from 0 to 48 h in IMR-32 cell lines. **(N)** Scratch assay results of ALDOC knockdown compared to control group in SH-SY5Y cell lines. **(O)** Statistical analysis of cell migration rate from 0 to 48 h in SH-SY5Y cell lines. **(P)** Transwell assay results of ALDOC knockdown compared to control group in IMR32 cell line. **(Q)** Statistical analysis of the number of migrated cells per field in Transwell assay of ALDOC knockdown compared to control group in IMR32 cell line. **(R)** Transwell assay results of ALDOC knockdown compared to control group in SH-SY5Y cell line. **(S)** Statistical analysis of the number of migrated cells per field in Transwell assay of ALDOC knockdown compared to control group in SH-SY5Y cell line. The scratch scale: 500 μm; Transwell scale: 100 μm. (ns, No statistically significant; **, P<0.01; ***, P<0.001; ****, P<0.0001).

**Figure 4:**
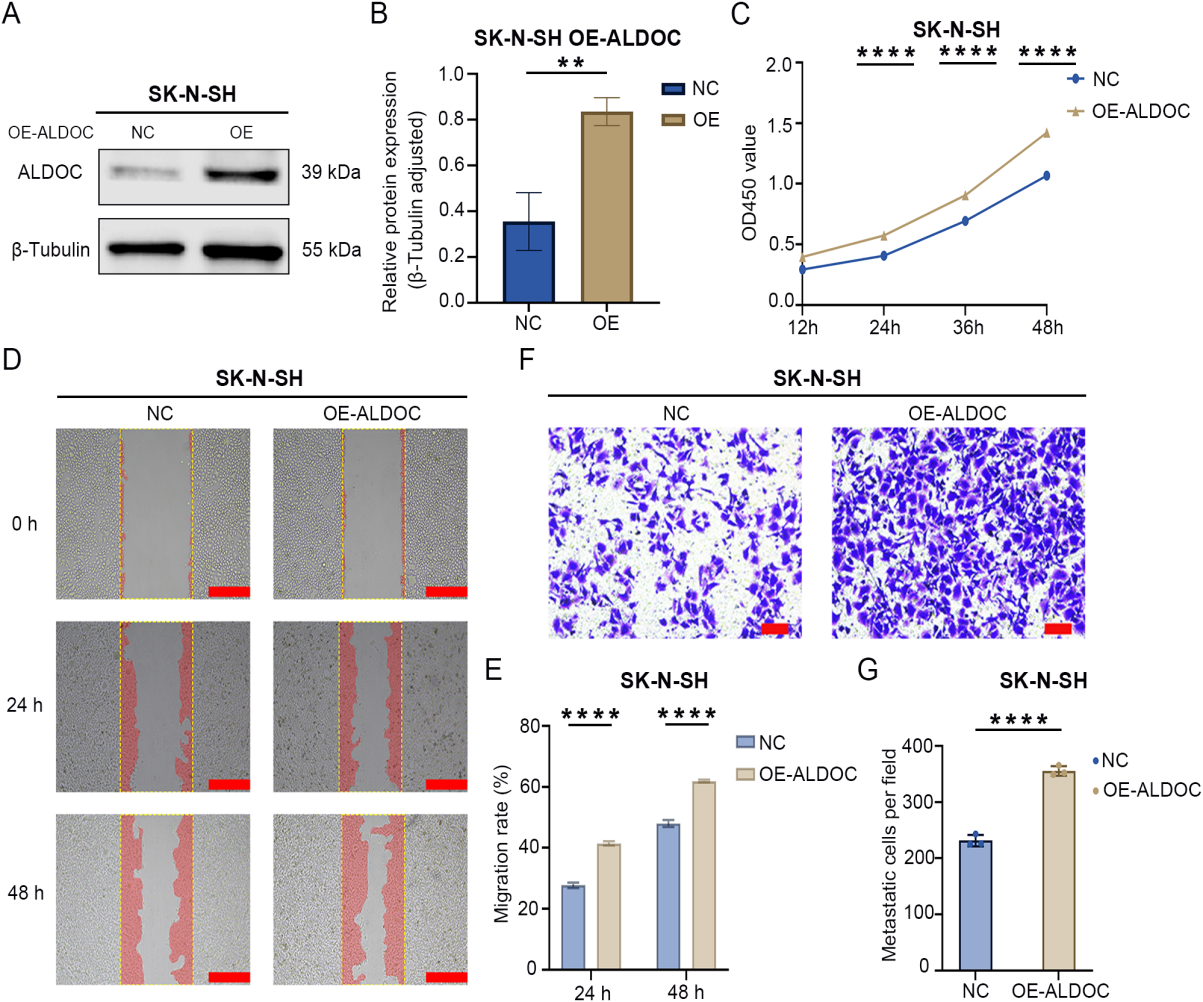
Overexpression of ALDOC promotes the proliferation and migration of NB cells. **(A)** Protein validation of ALDOC overexpression in SK-N-SHY cell line. **(B)** Statistical analysis of protein validation results of ALDOC overexpression in SK-N-SH cell line. **(C)** CCK8 proliferation assay results of ALDOC overexpression compared to control group in SK-N-SH cell line. **(D)** Scratch assay results of ALDOC overexpression compared to control group in SK-N-SH cell line. **(E)** Statistical analysis of cell migration rate from 0 to 48 hours in ALDOC overexpression compared to control group in SK-N-SH cell line. **(F)** Transwell assay results of ALDOC overexpression compared to control group in SK-N-SH cell line. **(G)** Statistical analysis of the number of migrated cells per field in Transwell assay of ALDOC overexpression compared to control group in SK-N-SH cell line. The scratch scale: 500 μm; Transwell scale: 100 μm. (**, P<0.01; ****, P<0.0001).

The experiments conducted in this study were repeated three or more times. The experimental data are presented as the mean ± standard errors of the mean (SEMs). GraphPad Prism 9.5 software was used for data processing and visualization. For the analysis of age and OS (overall survival time) in **Table 1**, we used the Wilcoxon test. The statistical analysis for gender and status was performed using the Chi-squared test, while the Fisher’s exact test was applied for the INSS stage. For MYCN status, Yates’ correction test was employed. Additionally, for comparisons between two groups, the t-test was used, as reflected in the statistical results in **Figures 1**–**4**. xxxThe significance levels used in the statistical analysis of the data are represented as follows in the graphs and tables: ns, no significant difference; *, P < 0.05; **, P < 0.01; ***, P < 0.001; and ****, P < 0.0001. Unless specified otherwise, P < 0.05 was considered to indicate statistical significance.

## 3 Results

### 3.1 Screening of key genes related to aerobic glycolysis in NB via histological analysis

We selected data from the GEO dataset GSE49710 for the differential analysis using limma to eliminate inter-sample differences, identifying upregulated and downregulated genes between stage 4 and non-stage 4 patients (**Figure 1A**). The obtained differentially expressed-genes (DEGs) were subjected to Kyoto Encyclopedia of Genes and Genomes (KEGG) and Gene Ontology (GO) functional enrichment analyses, which revealed enrichment in metabolism-related pathways, especially in glycolysis/gluconeogenesis (**Figures 1B, C**). We subsequently downloaded glycolysis-related genes from the Molecular Signatures Database (MSigDB) website and used online Venn diagram tools to intersect them with the DEGs, thus identifying 9 glycolysis-related differentially expressed genes (GRGs) (**Figure 1D**).

Then, we applied LASSO-Cox regression modeling to the GRGs to screen for prognostic genes. When the median of the sum of squared residuals was minimized, λ was selected to establish a risk scoring model (**Figures 1E, F**). The risk score of the Lasso-Cox regression analysis was =-0.0717777246765648*ALDOC+0.562603992797114*CENPA-0.615340599943134*ALDH3A2-0.0216167041592148*MERTK-0.125752365027469*CD44. We obtained a signature consisting of 5 genes, namely, ALDOC, CENPA, ALDH3A2, MERTK, and CD44. We divided the training set into high and low-risk groups based on the risk score using the median cutoff value and analyzed the differences in prognostic scores between the groups. **Figure 1G** displays the risk distribution, survival status, and gene expression patterns in the training cohort, indicating a greater probability of early death in the high-risk group. According to the overall K-M curve (**Figure 1H**), the OS of the high-risk group was significantly lower than that of the low-medium-risk group, suggesting a lower survival rate in the high-risk group. **Figure 1I** shows the time-dependent ROC curve for predicting OS in the training cohort, with AUCs of 0.84 at 1 year, 0.88 at 3 years, and 0.90 at 5 years, indicating that the model has good predictive ability.

### 3.2 determination of independent risk factors and verification of ALDOC

Next, we selected data from the Target database as a validation set to validate the prognostic model described above. The results are shown in **Figure 2**, in which the K-M curve results are consistent with those for the training set; the prognosis of the high-risk group was significantly worse (**Figure 2A**). Moreover, the AUCs at 1, 3, and 5 years were 0.54, 0.59, and 0.63, respectively (**Figure 2B**), indicating that the Lasso prognostic model we constructed has a good predictive ability for the NB prognosis. We then further analyzed the 5 genes in the prognostic model. First, we included the 5 DEGs related to glycolysis and clinicopathological parameters (age, gender, stage and grade) in the clinical prediction model. The results were intuitively displayed by drawing a nomogram (**Figure 2C**), and **Figure 2D** shows the calibration curves based on the nomogram for 1-, 3-, and 5-year survival. The closer the curve is to the standard curve, the better the predictive ability. The results revealed that only two genes, ALDOC and ALDH3A2, were statistically significant.

We Subsequently analyzed the whole-genome sequencing results of 15 clinical NB specimens to further investigate the two genes mentioned above. We first conducted an analysis at the mRNA level (still compared the stage 4 group and non-stage 4 group). According to the statistical analysis, the p-values for both genes were less than 0.05: the p-value for ALDOC was 0.00787326, whereas that for ALDH3A2 was 0.005594 (**Figure 2E**). Next, we conducted a protein-level analysis: the p-value for ALDOC was 0.007229673, whereas that for ALDH3A2 was 0.187200335, which was not statistically significant (**Figure 2F**). By integrating the above results, we identified ALDOC as a gene that serves as an independent risk factor for high-risk NB.

### 3.3 ALDOC expression is associated with NB progression

#### 3.3.1 The downregulation of ALDOC expression inhibits NB cell proliferation and migration

For further exploration of the expression of ALDOC in NB cells, we performed Western blotting (WB) to assess ALDOC expression in five NB cell lines and a control cell line. The results revealed that, except for SK-N-BE(2) cells, ALDOC expression was higher in the other NB cell lines than in the 293T cells (**Figures 3A, B**). We selected the IMR-32 and SH-SY5Y cell lines for ALDOC knockdown (**Figures 3C, E**) and selected the SK-N-SH cell line, which presented relatively low ALDOC expression, for ALDOC overexpression (**Figure 4A**). After comprehensive consideration, we decided to proceed with the third knockdown site (corresponding to lentivirus ID: 115919-1) for the IMR-32 cell treatment group and the second knockdown site (corresponding to lentivirus ID: 115918-1) for the SH-SY5Y cell treatment group. Detailed sequence information for the knockdown and overexpression lentiviruses can be found in **Supplementary Figures S1**, **S2**.

Next, we analyzed the impact of interfering with ALDOC expression on the function of NB cells. First, we assessed the effect of ALDOC knockdown on NB cell proliferation through colony formation and CCK8 assays. The CCK8 assay confirmed that the downregulation of ALDOC expression markedly reduced cell viability (**Figures 3G, H**). Similarly, the results of the colony formation assay revealed a significant reduction in the number of colonies formed by NB cells upon ALDOC knockdown compared with that in the control group (**Figures 3I-K**). We subsequently investigated the effect of interfering with ALDOC expression on NB cell migration via Transwell and scratch assays. The scratch assays revealed that the migration rate of NB cells decreased after ALDOC knockdown (**Figures 3L-O**). Additionally, the Transwell assay results indicated significantly less cell migration in the ALDOC knockdown group than in the control group (**Figures 3P-S**). Through a comprehensive analysis of the results from the proliferation and migration experiments, we concluded that the downregulation of ALDOC expression significantly inhibited NB cell proliferation and migration.

#### 3.3.2 ALDOC overexpression promotes the proliferation and migration of NB cells

The aforementioned results demonstrated that the downregulation of ALDOC expression can inhibit NB cell proliferation and migration. Next, we investigated the effect of ALDOC overexpression on NB cell function by constructing an ALDOC overexpression vector (**Figures 4A, B**). The CCK8 assay results revealed a significant increase in cell viability after ALDOC overexpression (**Figure 4C**). Moreover, the scratch assay results revealed a greater migration rate in the overexpression group (**Figures 4D, E**), and the Transwell assay results revealed a greater number of migrated cells in the overexpression group than in the control group (**Figures 4F, G**). Based on the aforementioned experiments, we concluded that ALDOC promotes NB cell proliferation and migration.

### 3.4 ALDOC regulates the rate of glycolysis, thereby influencing the progression of NB

#### 3.4.1 Downregulation of ALDOC expression can decrease the rate of glycolysis

**Figure 5:**
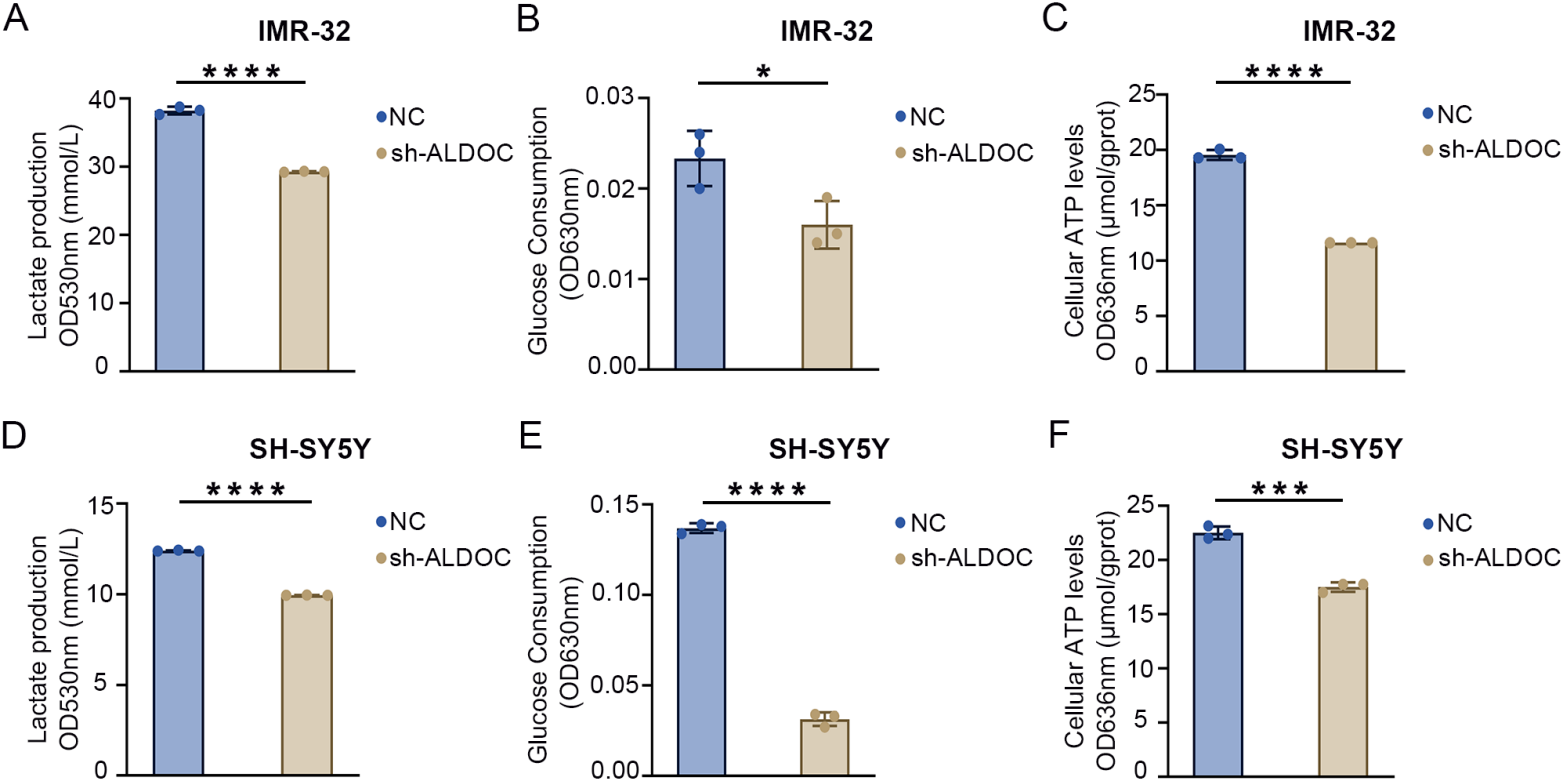
Downregulation of ALDOC expression can inhibit glycolysis rate. **(A)** Lactate production in ALDOC knockdown and control groups in IMR32 cell line. **(B)** Glucose absorption and utilization in ALDOC knockdown and control groups in IMR32 cell line. **(C)** ATP production in ALDOC knockdown and control groups in IMR32 cell line. **(D)** Lactate production in ALDOC knockdown and control groups in SH-SY5Y cell line. **(E)** Glucose absorption and utilization in ALDOC knockdown and control groups in SH-SY5Y cell line. **(F)** ATP production in ALDOC knockdown and control groups in SH-SY5Y cell line. (*, P<0.05; ***, P<0.001; ****, P<0.0001).

As established in the previous sections, ALDOC promotes NB cell proliferation and migration, and given the association between NB and glycolysis indicated in previous analyses, we investigated the effect of downregulating ALDOC expression on NB cell glycolysis. First, we assessed lactate (LD) production levels and found that compared with that in the control group, LD production in the ALDOC knockdown group was significantly lower (**Figures 5A, D**). Additionally, an analysis of glucose levels in the culture medium revealed a decrease in glucose uptake by NB cells after ALDOC knockdown (**Figures 5B, E**). The purpose of tumor cell glycolysis is to rapidly generate ATP and release energy to sustain the rapid proliferation of tumor cells. Therefore, we measured the ATP content in each cell line, and compared with those in the control group, the cells in the ALDOC knockdown group produced less ATP (**Figures 5C, F**). The consistent results of these three experiments indicate that interfering with ALDOC expression can decrease the glycolytic rate of NB cells.

#### 3.4.2 Overexpression of ALDOC promotes glycolysis and accelerates the rate of glycolysis

**Figure 6:**
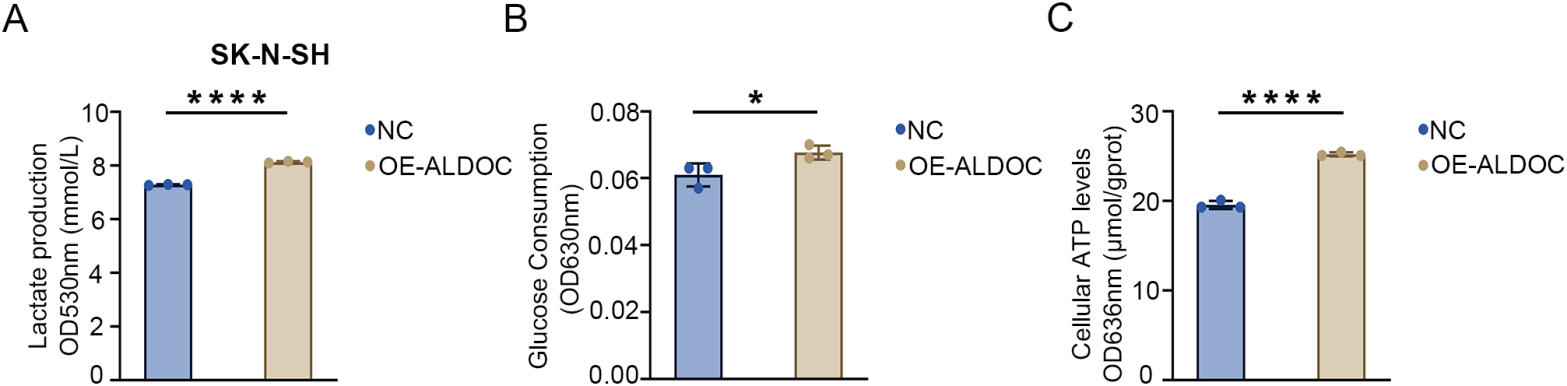
Overexpression of ALDOC promotes glycolysis and accelerates the rate of glycolysis. **(A)** Lactate production in the ALDOC overexpression group and control group in SK-N-SH cell line. **(B)** Glucose absorption and utilization in the ALDOC overexpression group and control group in SK-N-SH cell line. **(C)** ATP production in the ALDOC overexpression group and control group in SK-N-SH cell line. (*P<0.05; ****P<0.0001).

The results described in the previous section confirmed that knocking down ALDOC can decrease the glycolytic efficiency. Next, we analyzed the effect of ALDOC overexpression on NB cell glycolysis. A significant increase in LD production was observed after ALDOC overexpression (**Figure 6A**), indicating that ALDOC promoted glycolysis. In the glucose (GLU) detection experiment, an increase in glucose consumption was observed in the cell lines overexpressing ALDOC (**Figure 6B**). Finally, the ATP content was measured, and compared with the control cells, the ALDOC-overexpressing cells exhibited higher ATP production (**Figure 6C**). The results for the aforementioned glycolysis indicators obtained after ALDOC overexpression experimentally demonstrated that overexpressing ALDOC can increase glycolytic efficiency.

The aforementioned knockdown and overexpression experiments revealed that ALDOC promotes ATP production by increasing the rate of glycolysis, thereby providing energy for the rapid proliferation of NB cells. Combining these findings with those of previous experiments on cell functionality, we ultimately concluded that ALDOC likely promotes NB progression by increasing the glycolytic rate.

### 3.5 ALDOC affects sensitivity to cisplatin and cyclophosphamide

**Figure 7:**
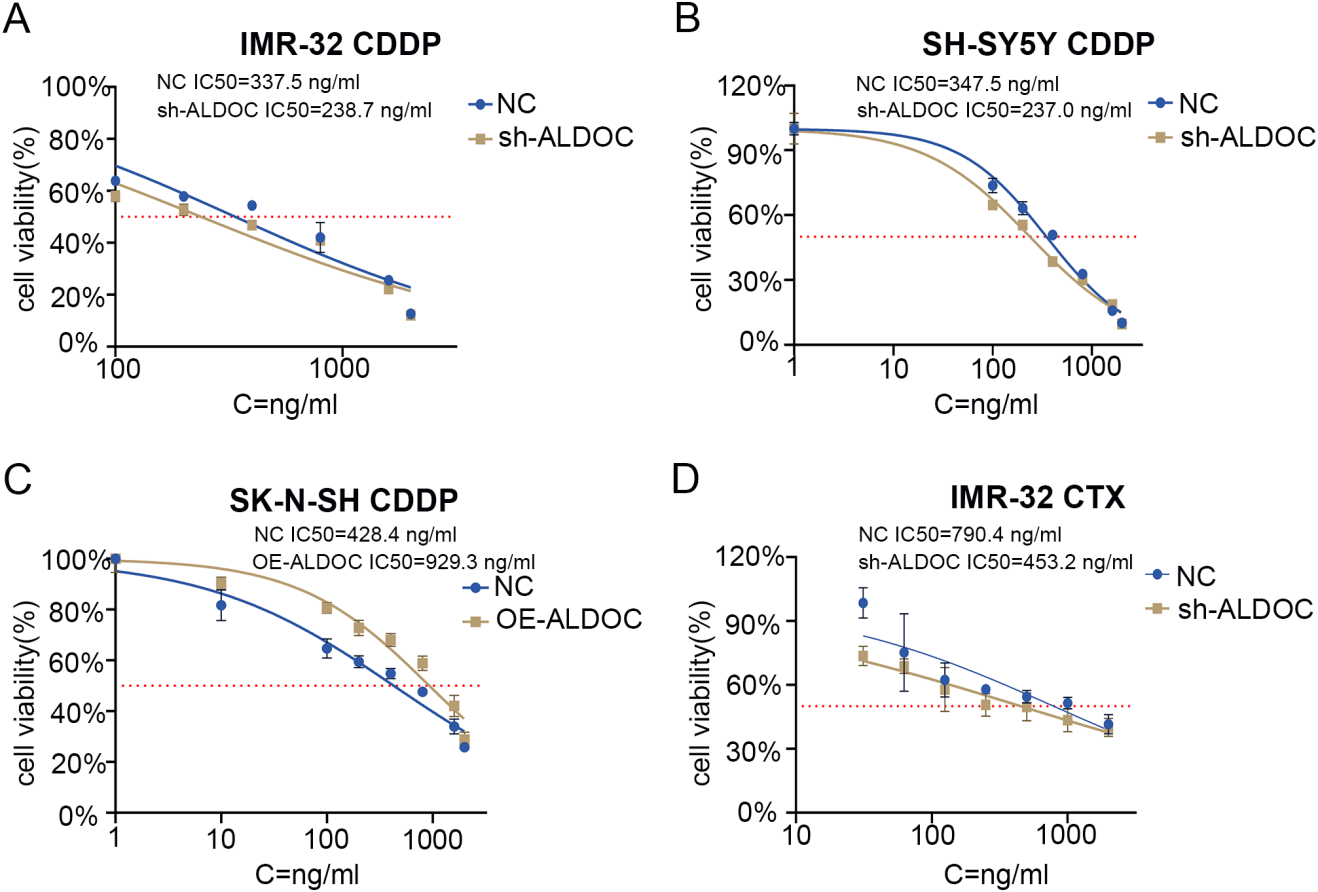
ALDOC affects the sensitivity of chemotherapy drugs. **(A)** IC50 value of cisplatin in control group vs. ALDOC knockdown group in IMR32 cell line. **(B)** IC50 value of cisplatin in control group vs. ALDOC knockdown group in SH-SY5Y cell line. **(C)** IC50 value of cisplatin in control group vs. ALDOC overexpression group in SK-N-SH cell line. **(D)** IC50 value of cyclophosphamide in control group vs. ALDOC knockdown group in IMR32 cell line. (CDDP, cisplatin; CTX, cyclophosphamide).

Based on the NB treatment guidelines (primarily based on the 2019 version in China) and analyses of small molecules and drugs that may interact with ALDOC (the detailed information regarding potential drugs and small-molecule compounds predicted to interact with ALDOC is provided in **Supplementary Figure S3**), we identified cisplatin as a commonly used drug in NB chemotherapy. Therefore, we chose cisplatin to analyze the changes in the sensitivity of NB cells to cisplatin after ALDOC knockdown/overexpression. Compared with the control group, the IC50 value of cisplatin decreased significantly after ALDOC knockdown (**Figures 7A, B**), whereas the IC50 value of cisplatin was significantly higher in cells overexpressing ALDOC (**Figure 7C**).

In clinical practice, the chemotherapy regimen for NB typically involves combination chemotherapy, especially in resource-limited areas, where regular chemotherapy is a feasible option for maintaining treatment (28). Therefore, we investigated another commonly used chemotherapeutic drug for NB treatment: cyclophosphamide. Compared with that in the control group, the IC50 value of cyclophosphamide was significantly lower in the ALDOC knockdown group (**Figure 7D**).

In summary, through the determination of the IC50 values for cisplatin and cyclophosphamide in NB cells with different ALDOC expression levels, we found that ALDOC can significantly affect the sensitivity of NB cells to commonly used chemotherapeutic drugs. Specifically, the expression level of ALDOC is inversely correlated with the sensitivity of NB cells to chemotherapy drugs. Based on these and previous experimental results, we believe that interfering with ALDOC expression not only inhibits the activity of NB cells but also increases their sensitivity to chemotherapy drugs. These findings have implications for subsequent treatment and drug development for this tumor type.

### 3.6 Downregulation of ALDOC expression can destabilize MYCN, leading to its ubiquitination and degradation

**Figure 8:**
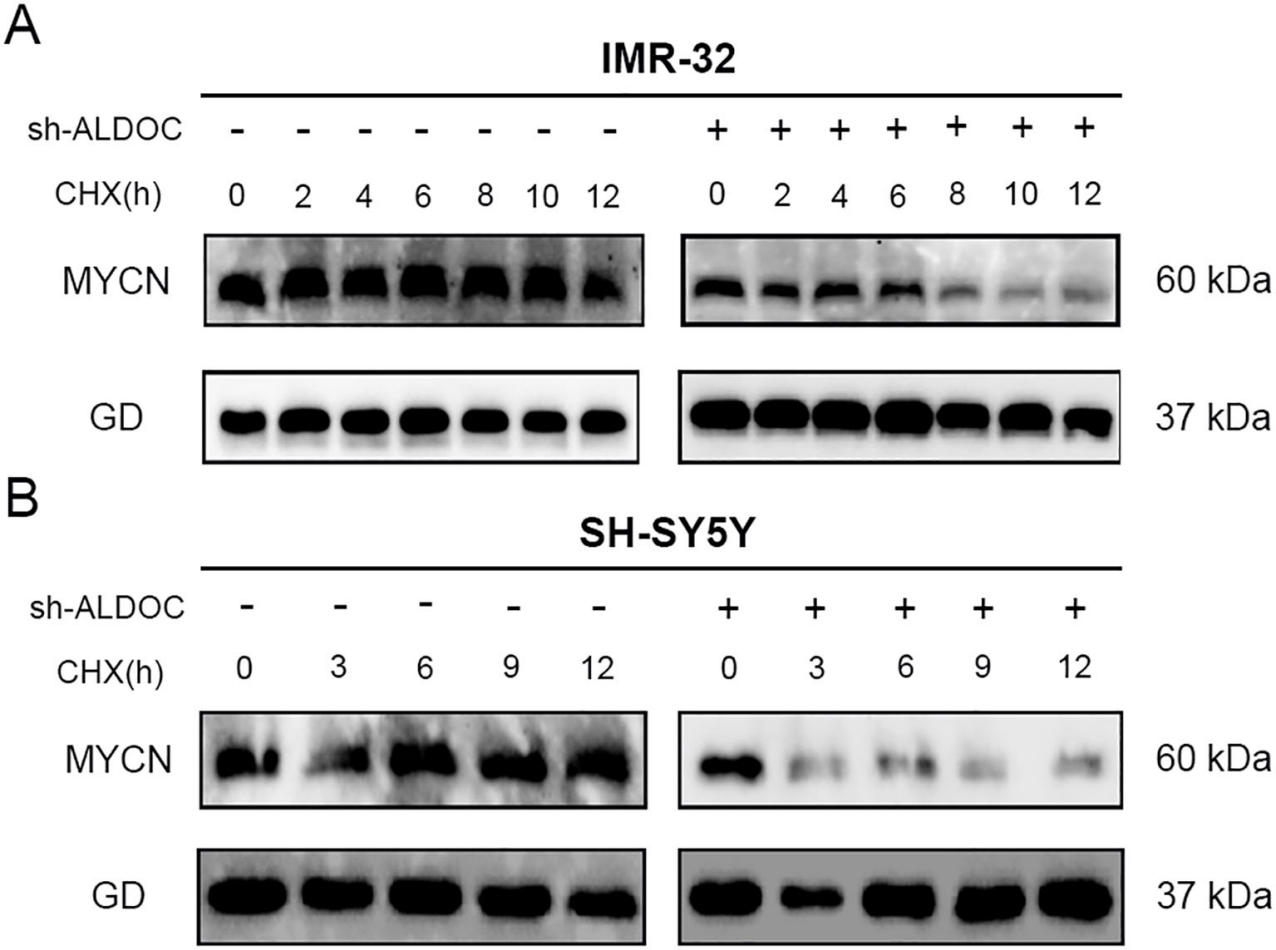
Downregulation of ALDOC expression can disrupt the stability of MYCN. **(A)** The expression of MYCN protein in the ALDOC knockdown group and control group in IMR32 cell line as CHX treatment time extends. **(B)** The expression of MYCN protein in the ALDOC knockdown group and control group in SH-SY5Y cell line as CHX treatment time extends. (GD: GAPDH, the housekeeping gene in this experiment).

Based on relevant literature reports, ALDOC likely plays a crucial role in maintaining the stability of MYCN (29–31). In our experiments, compared with that in the control group, the stability of the MYCN protein decreased gradually over time in ALDOC knockdown cell lines, as evidenced by the decreased expression of MYCN with increasing exposure to cycloheximide (CHX) (**Figures 8A, B**). These findings suggest that ALDOC knockdown affects the stability of MYCN, leading to its degradation over time.

**Figure 9:**
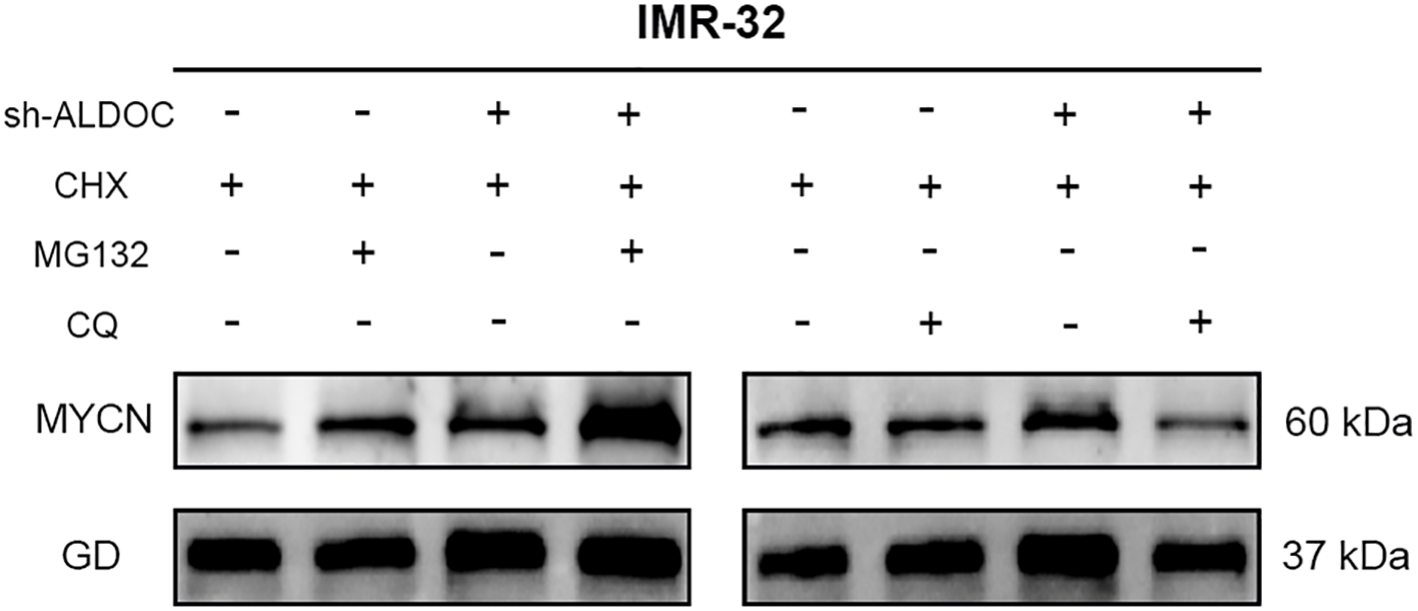
The MYCN protein undergoes degradation through the ubiquitin-proteasome system. (GD: GAPDH, the housekeeping gene in this experiment).

Research has demonstrated that the downregulation of ALDOC expression leads to the degradation of the MYCN protein. Therefore, our focus shifted to determining the degradation pathway for the MYCN protein in NB cells. We conducted experiments to analyze this phenomenon, and the results are presented in **Figure 9**. Compared with those in the control group, cells treated with MG132 presented significantly higher MYCN protein expression, whereas those treated with CQ presented no significant change and even a slight decreasing trend in MYCN protein expression. Therefore, we conclude that the degradation of MYCN is mediated by the ubiquitin-proteasome system.

In our experiment, we discovered a discrepancy with the expected results: after ALDOC knockdown, MYCN expression should have decreased accordingly, but this was not observed in **Figure 9**. Our research group hypothesizes that factors influencing MYCN expression are not limited to ALDOC alone. It is possible that changes in ALDOC lead to alterations in another cellular factor, resulting in an increase in MYCN. Additionally, upon reviewing previous experimental records, we found that our comparisons were based on internal groups: the control group with and without MG132, and the knockdown group with and without MG132. Finally, our group predicted that there might be MYCN binding sites in the ALDOC promoter region. Thus, after ALDOC knockdown, cells may compensate for the reduced ALDOC expression by increasing MYCN levels to obtain energy. However, due to current laboratory constraints, we cannot investigate the specific mechanisms in depth. We plan to conduct further research on this topic when conditions allow.

A comprehensive analysis revealed an association between the protein expression level of the ALDOC and the stability of MYCN protein—downregulation of ALDOC expression leads to the destabilization of MYCN, resulting in its degradation. In other words, the expression of ALDOC can maintain MYCN stability.

## 4 Discussion

In this study, we conducted a bioinformatics analysis to explore the differences in gene expression between patients with late-stage NB and early- to mid-stage NB. We found that ALDOC serves as an independent risk factor for high-risk NB. Furthermore, our sequencing results from pathology specimens revealed significant differences in the ALDOC mRNA and protein expression levels between stage 4 and non-stage 4 NB patients. Subsequently, we validated these findings through Western blot analysis, which revealed higher ALDOC expression levels in NB cells than in normal cells. Moreover, the downregulation of ALDOC expression significantly inhibited NB cell proliferation and migration, whereas the upregulation of ALDOC expression significantly enhanced NB cell proliferation and migration. Additionally, ALDOC knockdown led to a significant decrease in the IC50 values for cisplatin and cyclophosphamide in NB cells, indicating that ALDOC expression can influence NB cell sensitivity to these chemotherapeutic drugs. Furthermore, our LD, glucose, and ATP assays in NB cells revealed that the downregulation of ALDOC expression significantly inhibited glycolysis, whereas the overexpression of ALDOC increased glycolysis. Collectively, these experimental results suggest that ALDOC promotes NB progression by enhancing glycolysis, thereby reducing NB cell sensitivity to chemotherapy drugs.

Our findings are consistent with those of other researchers, indicating that interference with ALDOC expression can inhibit glycolysis and consequently suppress tumor growth. These findings have been confirmed in various studies on different types of tumors. For example, the downregulation of ALDOC expression inhibits glycolysis and proliferation in gallbladder cancer (GBC) cells (32), whereas reduced ALDOC expression weakens glycolysis and inhibits breast cancer growth (33). Our experimental results represent the first report of ALDOC in NB. Findings from other tumors suggest that ALDOC not only influences NB cell proliferation through glycolysis but also affects NB cell sensitivity to chemotherapy drugs. We observed an increase in NADPH expression in NB cells following ALDOC overexpression (**Supplementary Figure S4**), a process that is related to the mechanism discussed in the Introduction. Tumor cells produce large amounts of NADPH to maintain GSH activity and reduce sensitivity to chemotherapy drugs. In the field of cancer research, cancer is increasingly recognized as a metabolic disease involving disturbances in the metabolism of glucose, glutamine, and ketone bodies. In particular, energy metabolism fueled by glucose has been reported to be a characteristic of tumor progression (34, 35). Thus, targeting tumor metabolism to kill cancer cells may represent an effective approach for treating malignant tumors.

One important reason for the poor treatment outcomes in high-risk NB patients is the occurrence of chemotherapy resistance (36, 37). Studies have shown that the dysregulation of glycolysis can alter tumor cell sensitivity to certain chemotherapy drugs, leading to chemotherapy resistance and consequently reducing treatment efficacy (38–42). Compared with most studies using single-agent therapies, in this study, we employed multiple drugs to assess differences in the sensitivity of NB cells with varying ALDOC expression levels. Our findings indicate that interference with ALDOC expression significantly reduces the IC50 values for cisplatin and cyclophosphamide in NB cells, whereas ALDOC overexpression leads to a significant increase in the IC50 values for these drugs. These findings suggest that the inhibition or downregulation of ALDOC protein expression can increase NB cell sensitivity to various chemotherapy drugs, thereby allowing them toexert therapeutic effects at lower doses. Our experimental results provide evidence that ALDOC could be a novel therapeutic target for NB treatment or reversing chemotherapy resistance, providing a new approach for the treatment of high-risk NB patients.

In addition to its involvement in regulating the glycolysis pathway, ALDOC can interact with glycogen synthase kinase-3β (GSK-3β) to activate the Wnt pathway. Therefore, ALDOC can serve as a modulator of Wnt signaling, stabilizing β-catenin and facilitating its translocation into the nucleus to activate downstream factors, thereby contributing to tumorigenesis. Additionally, studies have shown that GSK3β can promote the degradation of MYC through phosphorylation (43–45), and that, ALDOC can interact with GSK3β and inhibit its activity (31), thereby preventing GSK3β from degrading MYC and maintaining MYC stability. MYCN is considered a driver of NB cell growth, and its amplification or overexpression is associated with a poor prognosis (7, 46–48). Research indicates that the downregulation of MYCN affects the levels and activity of glycolysis-related enzymes, and that patients with high MYCN expression have lower survival rates (49, 50). On the basis of our research findings and other literature reports, we utilized transcription factor prediction tools and found that MYC serves as a transcription factor for ALDOC. Therefore, the promoter region of ALDOC may contain binding sites for the MYC family member MYCN (**Supplementary Figure S5**). These findings suggest that MYCN may transcriptionally regulate ALDOC expression. Through experiments, we found that the downregulation of ALDOC expression disrupts MYCN stability, leading to its degradation. Therefore, we believe that the interaction between ALDOC and GSK3β can maintain the stability of MYCN, and that stable MYCN can activate ALDOC transcription, forming a malignant ALDOC-MYCN-ALDOC loop. This positive feedback loop plays a crucial role in the rapid progression of NB.

Although we have shown that ALDOC can promote the progression of NB by increasing the rate of glycolysis in NB cells and inhibiting the degradation of MYCN, stabilizing its oncogenic function and accelerating the malignant progression of NB, our research findings are based on cellular-level studies. Unfortunately, due to the lack of an animal facility in our laboratory, we did not validate our results at the animal level. Currently, our laboratory is not equipped with the necessary RT-qPCR instruments, so we are unable to measure the gene expression levels in the knockdown and overexpressed cells. This is a limitation of our current experiment. Furthermore, our laboratory lacks a Seahorse XF, so we are unable to conduct related metabolic analyses using this equipment. Instead, we rely on corresponding assay kits to measure metabolic indicators. Meanwhile, the MYCN antibody used in this experiment is only suitable for western blotting and cannot be used for IP experiments. Therefore, due to our current laboratory limitations, we are unable to verify the interaction between ALDOC and MYCN experimentally and can only rely on predictive methods. Additionally, our laboratory currently lacks the means to detect ubiquitin, so we are unable to conduct subsequent ubiquitin-related experiments. All above facts are regrettable, therefore, further investigations will be pursued when conditions allow in the future.

## 5 Conclusion

In summary, through this study, we primarily elucidated the potential mechanisms by which ALDOC promotes NB progression through the regulation of the glycolysis rate and maintenance of MYCN stability. Additionally, we determined how the expression level of ALDOC influences NB sensitivity to cisplatin and cyclophosphamide. These findings provide new insights for further research on NB progression and subsequent guidance for NB treatment and the development of new therapeutic agents.

## Data Availability

Existing datasets are available in a publicly accessible repository: Publicly available datasets were analyzed in this study. This data can be found here: GSE49710: https://www.ncbi.nlm.nih.gov/geo/query/acc.cgi?acc=GSE49710; GSE62564: https://www.ncbi.nlm.nih.gov/geo/query/acc.cgi?acc=GSE62564; Target: https://www.cancer.gov/ccg/research/genome-sequencing/target/studied-cancers/neuroblastoma.

## Supplementary material

Supplementary Figure 1 Sequence information of lentiviral knockdown sites and corresponding vector map.

Supplementary Figure 2 Sequence information for lentiviral overexpression sites and corresponding vector map.

Supplementary Figure 3 Prediction of Small Molecule Compounds and Drugs Targeting ALDOC. The red box in this figure is successful prediction of the drug cisplatin.

Supplementary Figure 4 The protein expression levels of NADPH in SK-N-SH cells. (GD: GAPDH, the housekeeping gene in this experiment).

Supplementary Figure 5 Prediction of ALDOC Transcription Factors. (The green annotations represent mRNA, while the blue annotations represent miRNA).

## References

[1] Mlakar V Jurkovic Mlakar S Lopez G Maris JM Ansari M Gumy-Pause F. 11q deletion in neuroblastoma: a review of biological and clinical implications. Mol Cancer. (2017) 16. doi: 10.1186/s12943-017-0686-8 PMC5492892 28662712

[2] Twist CJ Schmidt ML Naranjo A London WB Tenney SC Marachelian A. Maintaining outstanding outcomes using response- and biology-based therapy for intermediate-risk neuroblastoma: A report from the children’s oncology group study ANBL0531. J Clin Oncol. (2019) 37:3243–55. doi: 10.1200/JCO.19.00919 PMC6881103 31386611

[3] Maris JM. Recent advances in neuroblastoma. N Engl J Med. (2010) 362:2202–11. doi: 10.1056/NEJMra0804577 PMC3306838 20558371

[4] Alaggio R. WHO classification of tumours: paediatric tumours. Int Agency Res Cancer. (2022).

[5] London WB Castleberry RP Matthay KK Look AT Seeger RC Shimada H. Evidence for an age cutoff greater than 365 days for neuroblastoma risk group stratification in the children’s oncology group. J Clin Oncol. (2005) 23:6459–65. doi: 10.1200/JCO.2005.05.571 16116153

[6] Yamamoto K Hanada R Kikuchi A Ichikawa M Aihara T Oguma E. Spontaneous regression of localized neuroblastoma detected by mass screening. J Clin Oncol. (1998) 16:1265–9. doi: 10.1200/JCO.1998.16.4.1265 9552024

[7] Maris JM Hogarty MD Bagatell R Cohn SL. Neuroblastoma. Lancet. (2007) 369:2106–20. doi: 10.1016/S0140-6736(07)60983-0 17586306

[8] Weilbaecher KN Guise TA McCauley LK. Cancer to bone: a fatal attraction. Nat Rev Cancer. (2011) 11:411–25. doi: 10.1038/nrc3055 PMC3666847 21593787

[9] Hochheuser C Windt LJ Kunze NY de Vos DL Tytgat GAM Voermans C. Mesenchymal stromal cells in neuroblastoma: exploring crosstalk and therapeutic implications. Stem Cells Dev. (2021) 30:59–78. doi: 10.1089/scd.2020.0142 33287630 PMC7826431

[10] DuBois SG Kalika Y Lukens JN Brodeur GM Seeger RC Atkinson JB. Metastatic sites in stage IV and IVS neuroblastoma correlate with age, tumor biology, and survival. J Pediatr Hematol/Oncol. (1999) 21:181–9. doi: 10.1097/00043426-199905000-00005 10363850

[11] Matthay KK Maris JM Schleiermacher G Nakagawara A Mackall CL Diller L. Neuroblastoma. Nat Rev Dis Primers. (2016) 2:16078. doi: 10.1038/nrdp.2016.78 27830764

[12] Louis CU Shohet JM. Neuroblastoma: molecular pathogenesis and therapy. Annu Rev Med. (2015) 66:49–63. doi: 10.1146/annurev-med-011514-023121 25386934 PMC4418018

[13] Zafar A Wang W Liu G Wang X Xian W McKeon F. Molecular targeting therapies for neuroblastoma: Progress and challenges. Medicinal Res Rev. (2020) 41:961–1021. doi: 10.1002/med.21750 PMC7906923 33155698

[14] Sait S Modak S. Anti-GD2 immunotherapy for neuroblastoma. Expert Rev Anticancer Ther. (2017) 17:889–904. doi: 10.1080/14737140.2017.1364995 28780888 PMC6082365

[15] Karami Fath M Bagherzadeh Torbati SM Saqagandomabadi V Yousefi Afshar O Khalilzad M Abedi S. The therapeutic effect of MSCs and their extracellular vesicles on neuroblastoma. Prog Biophys Mol Biol. (2024) 187:51–60. doi: 10.1016/j.pbiomolbio.2024.02.004 38373516

[16] Corallo D Dalla Vecchia M Lazic D Taschner-Mandl S Biffi A Aveic S. A molecular basis of tumor metastasis and current approaches to decode targeted migration-promoting events in pediatric neuroblastoma. Biochem Pharmacol. (2023) 215:115696. doi: 10.1016/j.bcp.2023.115696 37481138

[17] Zheng YL Li L Jia YX Zhang BZ Li JC Zhu YH. LINC01554-mediated glucose metabolism reprogramming suppresses tumorigenicity in hepatocellular carcinoma via downregulating PKM2 expression and inhibiting Akt/mTOR signaling pathway. Theranostics. (2019) 9:796–810. doi: 10.7150/thno.28992 30809309 PMC6376468

[18] Warburg O. On the origin of cancer cells. Science. (1956) 123:309–14. doi: 10.1126/science.123.3191.309 13298683

[19] Chen M Sheng XJ Qin YY Zhu S Wu QX Jia L. TBC1D8 amplification drives tumorigenesis through metabolism reprogramming in ovarian cancer. Theranostics. (2019) 9:676–90. doi: 10.7150/thno.30224 PMC6376479 30809301

[20] Ciavardelli D Rossi C Barcaroli D Volpe S Consalvo A Zucchelli M. Breast cancer stem cells rely on fermentative glycolysis and are sensitive to 2-deoxyglucose treatment. Cell Death Dis. (2014) 5:e1336–6. doi: 10.1038/cddis.2014.285 PMC4123079 25032859

[21] Shulkin BL Mitchell DS Ungar DR Prakash D Dole MG Castle VP. Neoplasms in a pediatric population: 2-[F-18]-fluoro-2-deoxy-D-glucose PET studies. Radiology. (1995) 194:495–500. doi: 10.1148/radiology.194.2.7824731 7824731

[22] Nilsson H Lindgren D Mandahl Forsberg A Mulder H Axelson H and Johansson ME. Primary clear cell renal carcinoma cells display minimal mitochondrial respiratory capacity resulting in pronounced sensitivity to glycolytic inhibition by 3-Bromopyruvate. Cell Death Dis. (2015) 6:e1585–5. doi: 10.1038/cddis.2014.545 PMC4669744 25569102

[23] Kim J-W Gao P Liu Y-C Semenza GL Dang CV. Hypoxia-inducible factor 1 and dysregulated c-myc cooperatively induce vascular endothelial growth factor and metabolic switches hexokinase 2 and pyruvate dehydrogenase kinase 1. Mol Cell Biol. (2023) 27:7381–93. doi: 10.1128/MCB.00440-07 PMC2169056 17785433

[24] Schwartzenberg-Bar-Yoseph F Armoni M Karnieli E. The tumor suppressor p53 down-regulates glucose transporters GLUT1 and GLUT4 gene expression. Cancer Res. (2004) 64:2627–33. doi: 10.1158/0008-5472.CAN-03-0846 15059920

[25] Ishikawa A Shiwa Y Katsuya N Maruyama R Fukui T Kuraoka K. Fructose-bisphosphate aldolase C expression is associated with poor prognosis and stemness in gastric cancer. Acta Histochem Et Cytochem. (2024) 57:165–74. doi: 10.1267/ahc.24-00044 PMC11565221 39552933

[26] Maruyama R Nagaoka Y Ishikawa A Akabane S Fujiki Y Taniyama D. Overexpression of aldolase, fructose-bisphosphate C and its association with spheroid formation in colorectal cancer. Pathol Int. (2022) 72:176–86. doi: 10.1111/pin.13200 35147255

[27] Tang F Cui Q. Diverse roles of aldolase enzymes in cancer development, drug resistance and therapeutic approaches as moonlighting enzymes. Med Oncol. (2024) 41:224. doi: 10.1007/s12032-024-02470-x 39120781

[28] Chou S-W Chang H-H. Evolution and contemporary role of metronomic chemotherapy in the treatment of neuroblastoma. Cancer Lett. (2024) 588. doi: 10.1016/j.canlet.2024.216617 38311055

[29] Chang YC Yang YC Tien CP Yang CJ Hsiao M. Roles of aldolase family genes in human cancers and diseases. Trends Endocrinol Metab. (2018) 29:549–59. doi: 10.1016/j.tem.2018.05.003 29907340

[30] Shang B Lu F Jiang S Xing M Mao X Yang G. ALDOC promotes non-small cell lung cancer through affecting MYC-mediated UBE2N transcription and regulating Wnt/beta-catenin pathway. Aging (Albany NY). (2023) 15:9614–32. doi: 10.18632/aging.205038 PMC10564444 37724906

[31] Caspi M Perry G Skalka N Meisel S Firsow A Amit M. Aldolase positively regulates of the canonical Wnt signaling pathway. Mol Cancer. (2014) 13. doi: 10.1186/1476-4598-13-164 PMC4094682 24993527

[32] Fan K Wang J Sun W Shen S Ni X Gong Z. MUC16 C-terminal binding with ALDOC disrupts the ability of ALDOC to sense glucose and promotes gallbladder carcinoma growth. Exp Cell Res. (2020) 394. doi: 10.1016/j.yexcr.2020.112118 32502493

[33] Yan L Wu M Wang T Yuan H Zhang X Zhang H. Breast cancer stem cells secrete MIF to mediate tumor metabolic reprogramming that drives immune evasion. Cancer Res. (2024) 84(8):1270–85. doi: 10.1158/0008-5472.c.7181269.v2 38335272

[34] Faubert B Solmonson A. and DeBerardinis RJ: Metabolic reprogramming and cancer progression. Science. (2020) 368. doi: 10.1126/science.aaw5473 PMC7227780 32273439

[35] Reinfeld BI Rathmell WK Kim TK Rathmell JC. The therapeutic implications of immunosuppressive tumor aerobic glycolysis. Cell Mol Immunol. (2021) 19:46–58. doi: 10.1038/s41423-021-00727-3 34239083 PMC8752729

[36] Pearson ADJ Pinkerton CR Lewis IJ Imeson J Ellershaw C Machin D. High-dose rapid and standard induction chemotherapy for patients aged over 1 year with stage 4 neuroblastoma: a randomised trial. Lancet Oncol. (2008) 9:247–56. doi: 10.1016/S1470-2045(08)70069-X 18308250

[37] Matthay KK Reynolds CP Seeger RC Shimada H Adkins ES Haas-Kogan D. Long-term results for children with high-risk neuroblastoma treated on a randomized trial of myeloablative therapy followed by 13-cis-retinoic acid: A children’s oncology group study. J Clin Oncol. (2009) 27:1007–13. doi: 10.1200/JCO.2007.13.8925 PMC2738615 19171716

[38] Chen J-Q Russo J. Dysregulation of glucose transport, glycolysis, TCA cycle and glutaminolysis by oncogenes and tumor suppressors in cancer cells. Biochim Biophys Acta (BBA) Reviews Cancer. (2012) 1826:370–84. doi: 10.1016/j.bbcan.2012.06.004 22750268

[39] Hamanaka RB Chandel NS. Targeting glucose metabolism for cancer therapy. J Exp Med. (2012) 209:211–5. doi: 10.1084/jem.20120162 PMC3280882 22330683

[40] Icard P Shulman S Farhat D Steyaert JM Alifano M Lincet H. How the Warburg effect supports aggressiveness and drug resistance of cancer cells? Drug Resist Update. (2018) 38:1–11. doi: 10.1016/j.drup.2018.03.001 29857814

[41] Marcucci F Rumio C. Glycolysis-induced drug resistance in tumors—A response to danger signals? Neoplasia. (2021) 23:234–45. doi: 10.1016/j.neo.2020.12.009 PMC7804361 33418276

[42] Xu RH Pelicano H Zhou Y Carew JS Feng L Bhalla KN. Inhibition of glycolysis in cancer cells: a novel strategy to overcome drug resistance associated with mitochondrial respiratory defect and hypoxia. Cancer Res. (2005) 65:613–21. doi: 10.1158/0008-5472.613.65.2 15695406

[43] Sears R Nuckolls F Haura E Taya Y Tamai K Nevins JR. Multiple Ras-dependent phosphorylation pathways regulate Myc protein stability. Genes Dev. (2000) 14:2501–14. doi: 10.1101/gad.836800 PMC316970 11018017

[44] Gregory MA Qi Y Hann SR. Phosphorylation by glycogen synthase kinase-3 controls c-myc proteolysis and subnuclear localization. J Biol Chem. (2003) 278:51606–12. doi: 10.1074/jbc.M310722200 14563837

[45] Yeh E Cunningham M Arnold H Chasse D Monteith T Ivaldi G. A signalling pathway controlling c-Myc degradation that impacts oncogenic transformation of human cells. Nat Cell Biol. (2004) 6:308–18. doi: 10.1038/ncb1110 15048125

[46] Nakata R Shimada H Fernandez GE Fanter R Fabbri M Malvar J. Contribution of neuroblastoma-derived exosomes to the production of pro-tumorigenic signals by bone marrow mesenchymal stromal cells. J Extracell Vesicles. (2017) 6:1332941. doi: 10.1080/20013078.2017.1332941 28717423 PMC5505006

[47] Fonseka P Liem M Ozcitti C Adda CG Ang CS Mathivanan S. Exosomes from N-Myc amplified neuroblastoma cells induce migration and confer chemoresistance to non-N-Myc amplified cells: implications of intra-tumour heterogeneity. J Extracell Vesicles. (2019) 8:1597614. doi: 10.1080/20013078.2019.1597614 31007876 PMC6461098

[48] Jansky S Sharma AK Körber V Quintero A Toprak UH Wecht EM. Single-cell transcriptomic analyses provide insights into the developmental origins of neuroblastoma. Nat Genet. (2021) 53:683–93. doi: 10.1038/s41588-021-00806-1 33767450

[49] Qing G Skuli N Mayes PA Pawel B Martinez D Maris JM. Combinatorial regulation of neuroblastoma tumor progression by N-Myc and hypoxia inducible factor HIF-1alpha. Cancer Res. (2010) 70:10351–61. doi: 10.1158/0008-5472.CAN-10-0740 PMC3005134 20961996

[50] Bishayee K Nazim UM Kumar V Kang J Kim J Huh SO. Reversing the HDAC-inhibitor mediated metabolic escape in MYCN-amplified neuroblastoma. Biomed Pharmacother. (2022) 150. doi: 10.1016/j.biopha.2022.113032 35486977

