# Supplementary figures and images for: ALDOC promotes neuroblastoma progression and modulates sensitivity to chemotherapy drugs by enhancing aerobic glycolysis
Source: Front Immunol. 2025 Apr 17;16:1573815. doi: 10.3389/fimmu.2025.1573815 (PMC12043483; doi:10.3389/fimmu.2025.1573815)

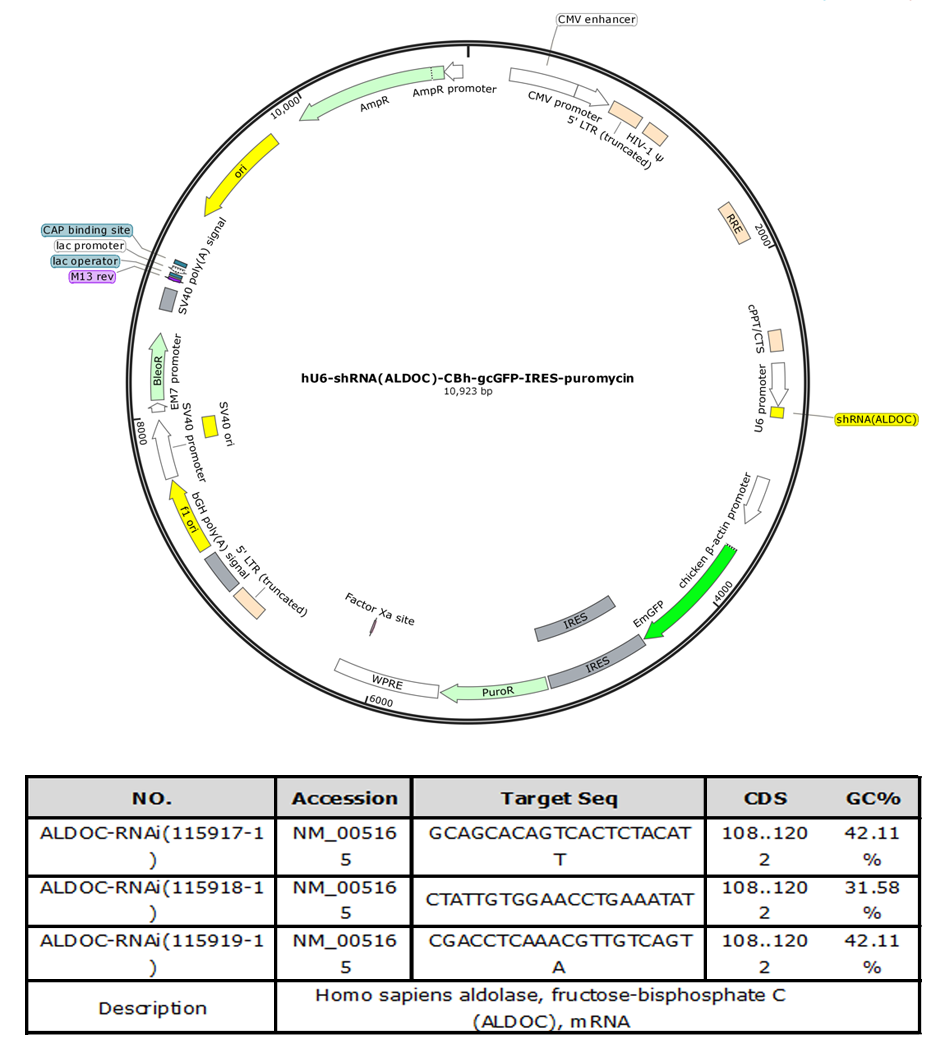

Supplement: Supplementary Figure 1 — Sequence information of lentiviral knockdown sites and corresponding vector map. [file Image1.tif]

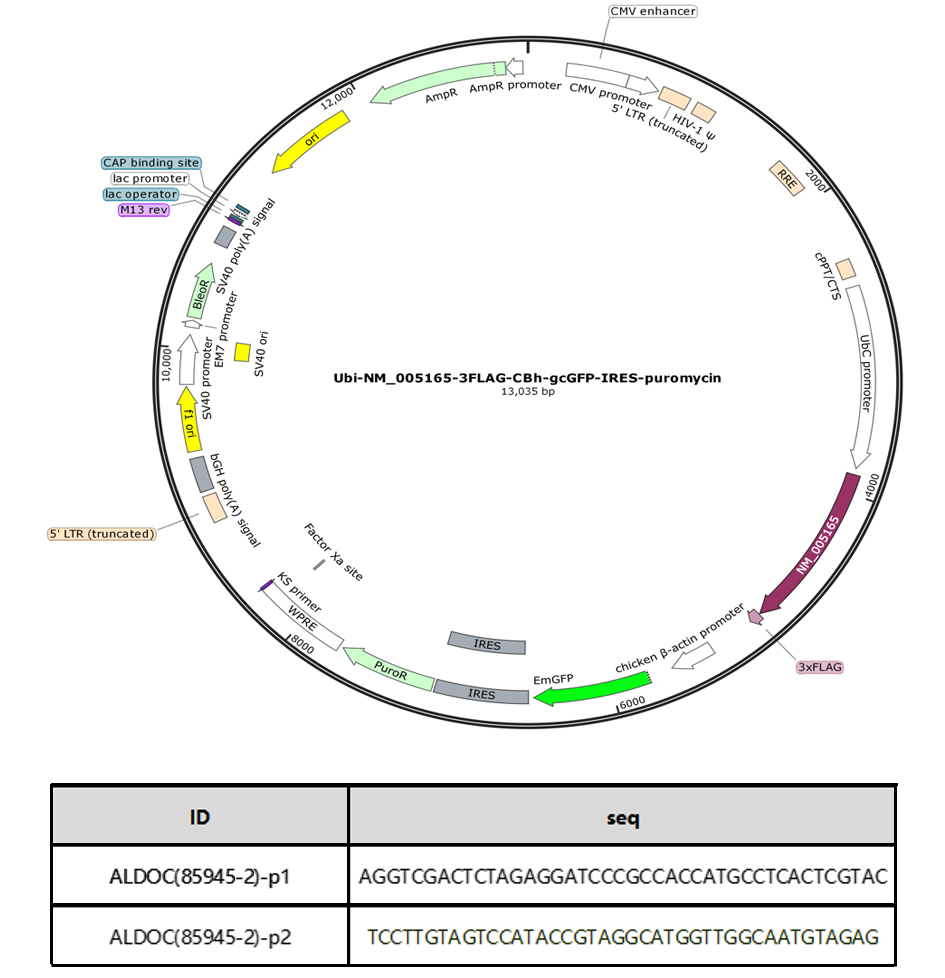

Supplement: Supplementary Figure 2 — Sequence information for lentiviral overexpression sites and corresponding vector map. [file Image2.tif]

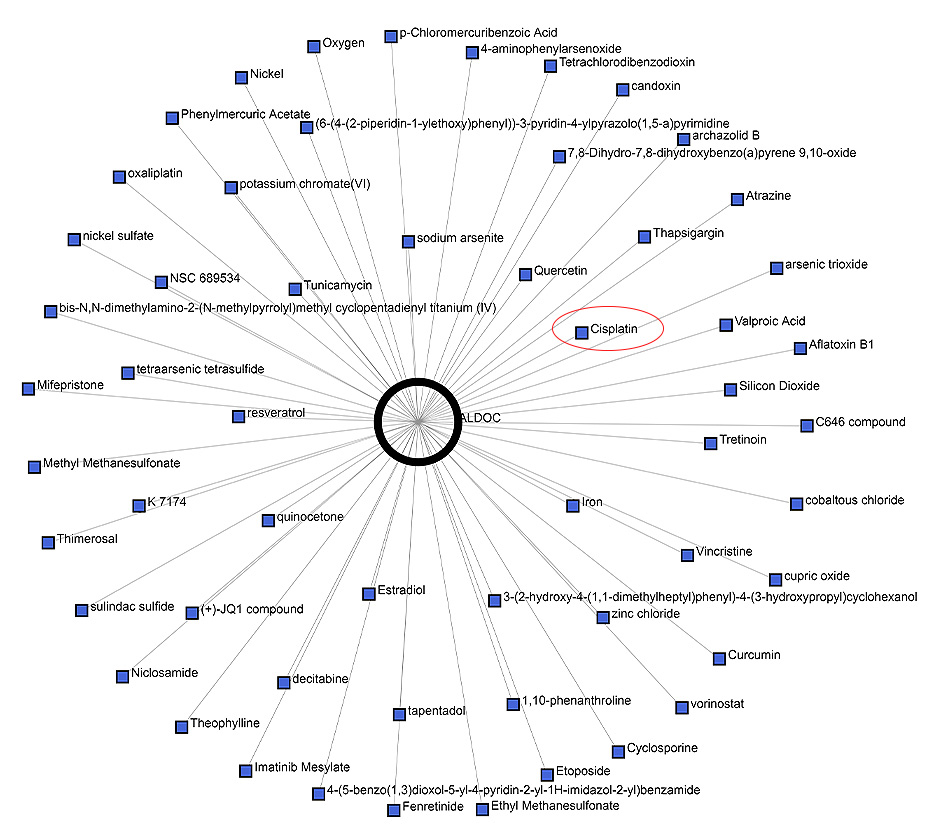

Supplement: Supplementary Figure 3 — Prediction of Small Molecule Compounds and Drugs Targeting ALDOC. The red box in this figure is successful prediction of the drug cisplatin. [file Image3.jpeg]

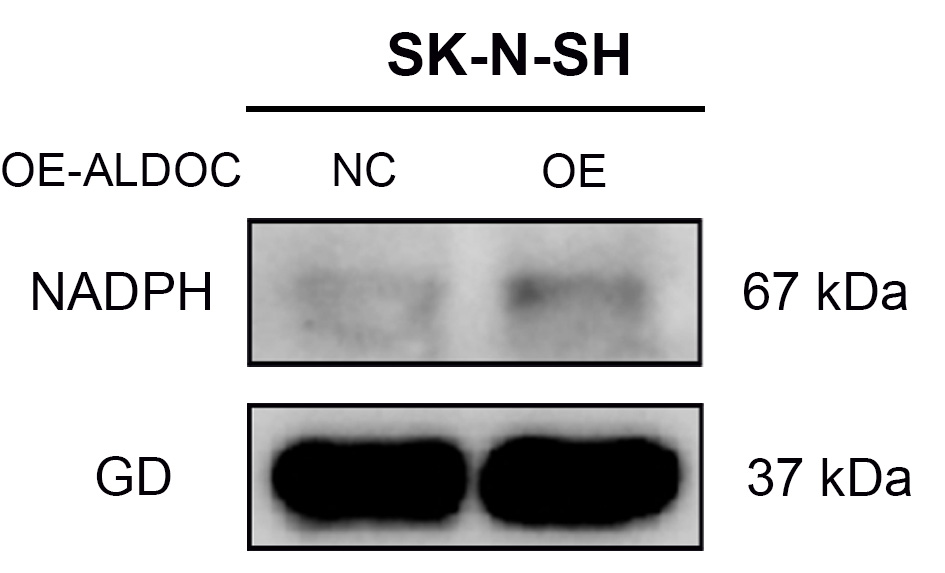

Supplement: Supplementary Figure 4 — The protein expression levels of NADPH in SK-N-SH cells. (GD: GAPDH, the housekeeping gene in this experiment). [file Image4.tif]

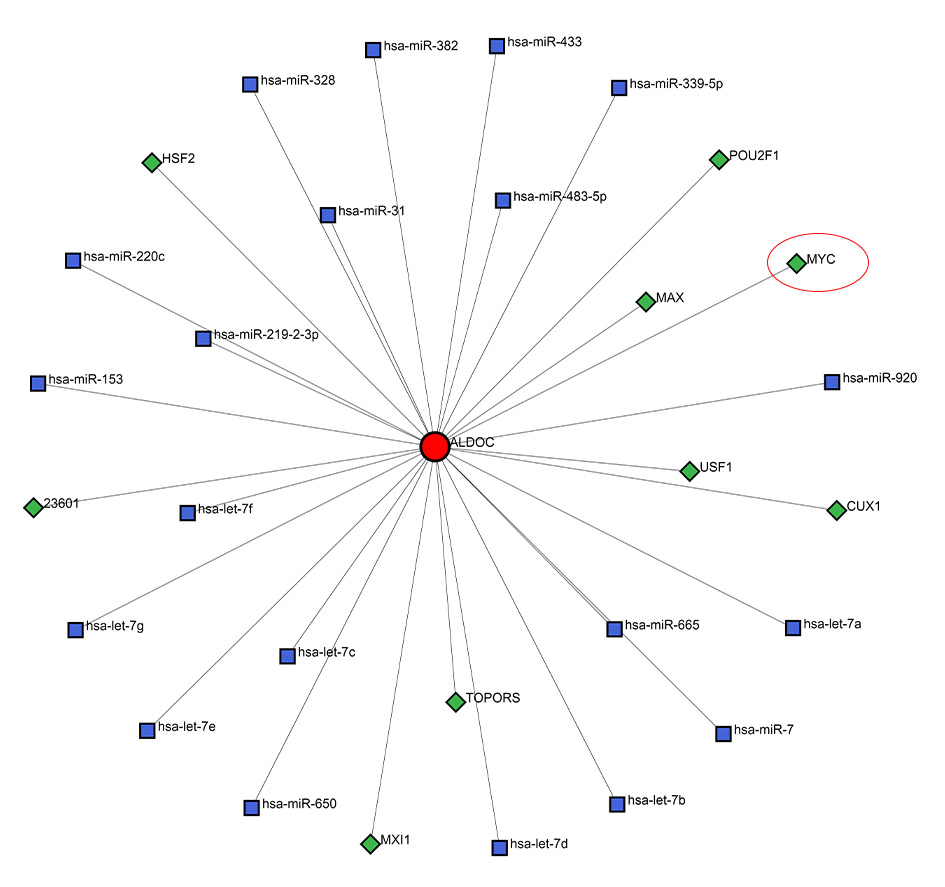

Supplement: Supplementary Figure 5 — Prediction of ALDOC Transcription Factors. (The green annotations represent mRNA, while the blue annotations represent miRNA). [file Image5.jpeg]
